# Prevalence of hypertension and associated cardiovascular risk factors in an urban slum in Nairobi, Kenya: A population-based survey

**DOI:** 10.1186/1471-2458-14-1177

**Published:** 2014-11-18

**Authors:** Mark David Joshi, Richard Ayah, Elijah Kaharo Njau, Rosemary Wanjiru, Joshua Kyateesa Kayima, Erastus Kennedy Njeru, Kenneth Kipyegon Mutai

**Affiliations:** Department of Clinical Medicine and Therapeutics, School of Medicine, College of Health Sciences, University of Nairobi, P.O. BOX 19676-00202, KNH Nairobi, Kenya; School of Public Health, College of Health Sciences, University of Nairobi, Nairobi, Kenya; University of Nairobi Partnership for Advanced Care and Treatment (PACT) – Centre of Excellence, Nairobi, Kenya

**Keywords:** Hypertension prevalence, Urban health, Poverty areas, Africa South of the Sahara, Non-communicable diseases

## Abstract

**Background:**

Urbanisation has been described as a key driver of the evolving non-communicable disease (NCD) epidemic. In Africa, hypertension is the commonest cardiovascular problem. We determined the prevalence and risk factor correlates of hypertension in the largest Nairobi slum.

**Methods:**

In 2010 we conducted a population-based household survey in Kibera, a large informal settlement in Nairobi City; utilising cluster sampling with probability proportional to size. Households were selected using a random walk method. The WHO instrument for stepwise surveillance (STEPS) of chronic disease risk factors was administered by trained medical assistants, who also recorded blood pressure (BP) and anthropometric measures. BP was recorded using a mercury sphygmomanometer utilising the American Heart Association guidelines. Hypertension was defined as per the 7th Report of the Joint National Committee or use of prescribed antihypertensive medication. Those with hypertension or with random capillary blood sugar (RCBS) >11.1 mmol/l had an 8 hours fasting venous blood sugar sample drawn. Age standardised prevalence was computed and multivariate analysis to assess associations.

**Results:**

We screened 2200 and enrolled 2061 adults; 50.9% were males; mean age was 33.4 years and 87% had primary level education. The age-standardised prevalence of hypertension (95% CI) was 22.8% (20.7, 24.9). 20% (53/258) were aware of their hypertensive status; 59.3% had pre-hypertension; 80% reported high levels of physical activity and 52% were classified as harmful alcohol drinkers; 10% were current smokers and 5% had diabetes. Majority of males had normal BMI and waist circumference, whereas a third of females were obese or overweight and 40% had central obesity. Older age, higher general and central obesity were independently associated with hypertension and higher SBP and DBP readings.

**Conclusions:**

Our findings of high prevalence of hypertension, in association with excess body weight in this poor urban slum community, point to the need for greater awareness and implementation of primary preventive strategies.

**Electronic supplementary material:**

The online version of this article (doi:10.1186/1471-2458-14-1177) contains supplementary material, which is available to authorized users.

## Background

The prevalence of Non-Communicable Diseases (NCD) in low- and middle-income countries is rising rapidly [1]. Half of the current health burden in developing nations is attributable to non-communicable diseases [2]. By 2020 it is projected that non-communicable diseases including, hypertension, will outstrip communicable diseases as the leading cause of death. Demographic changes, increasing urbanization and lifestyle changes are some of the factors contributing to the increasing burden of NCD [3].

Hypertension is a major contributor to NCD burden in both developed and developing countries [4]. Hypertension is a primary cause of haemorrhagic and atherothrombotic stroke, hypertensive heart disease, hypertensive kidney failure, coronary artery disease [5, 6].

Hypertension is now the most common cardiovascular problem in Africa, and it is estimated that more than 20 million people are affected [7]. In sub-Saharan Africa hypertensive end organ damage is a major source of morbidity and mortality [6]. The reported prevalence of hypertension in Africa ranges from 25% to 35% in adults aged 25 to 64 years [7], and increases with advancing age. A recent study of urban and rural Tanzania reported rates of stroke mortality higher than those of England and Wales, and suggested that untreated hypertension is an important etiological risk factor [8]. In Kenya there is a paucity of data on hypertension prevalence, and at the time of our survey in 2010 only two studies had been reported. The initial 1986 survey was undertaken in a regional centre, among both rural and urban residents, reported non-standardised hypertension prevalence of 6.4% [9]. A 2008 regional cross-sectional study, restricted to subjects aged over 50 yr and conducted in predominantly rural population, reported a prevalence of 50.1% [10].

Urbanisation is a key driver of the evolving NCD epidemic in developing countries [3]. In Kenya 22.3% of the population is urban, with an urban population growth rate of 4.2% almost double the national population growth rate of 2.4% [11]. In the capital city Nairobi, a majority (60%) of the population live in slums with much of the migrant population settling in slums. Furthermore 75% of the urban population growth is absorbed by informal settlements. To address the data void on the burden of hypertension in Kenya, we report on the prevalence and risk factor correlates of hypertension, in a representative sample of the rapidly expanding urban poor segment of our population.

## Methods

The urban slum of Kibera in Nairobi, the capital of Kenya was chosen as the study area. Kibera is located 5 km southwest of Nairobi city centre and is approximately 2.5 square kilometres, with an estimated 300,000 inhabitants [11]. This study was conducted using UN recommendation for cluster sampling with probability proportional to size adjusted to achieve our sample size [12]. A design effect of 2.0 was used to account for the clustering of the study participants. A sampling frame with the list of villages and the projected populations of each was obtained. As a result a total of 80 clusters each containing 10 households and thus 25 participants per cluster were created within the eight villages with the number of clusters proportional to the population size within the particular village. The households in each cluster were visited in a random walk method from the nearest health centre, church or school as the focal point in each cluster. Within each household, all consenting adults of 18 years and above, who had been residents of the area for more than 3 months, were enrolled. Exclusions included those under the age of 18 years; non-consenting individual and pregnant women. The next household was taken as the one nearest to that previously visited until the sample size for the given cluster was achieved. The WHO STEPS-wise approach for collecting surveillance data for non-communicable diseases (NCD’s) was adopted to collect data. The WHO STEP-wise approach to surveillance of non-communicable diseases is a validated instrument developed by WHO for collection of surveillance data on NCD’s in resource poor settings. It is a sequential process made up of three main sections, which are the risk stratification questionnaire (step1), anthropometric measurements (step 2) and biochemical measurements (step3) [13].

The survey was conducted between June and August 2010 with research teams visiting selected household between 8 am and 4 pm daily on weekdays. We minimized missing respondents by making a maximum of two revisits on subsequent Sundays. The questionnaire was administered by study trained registered medical assistant teams supervised by a study medical doctor. The questionnaire covered the smoking habits, alcohol use and physical activity pattern, history of prior evaluation for diabetes and hypertension, and if any, the medication and lifestyle counselling that had been given. Anthropometric measures were recorded as hereunder. Height was measured to the nearest 0.5 cm using a metal measuring tape against a wall and a flat headboard at right angles to the wall. Weight was measured to the nearest 100gms using a good quality bathroom scale (Ashton Meyers®) with the subject in light clothing and without shoes. Mid upper arm circumference was measured to the nearest 0.5 cm. Waist circumference was taken with a flexible tape measure placed on a horizontal plane at the level midpoint between the superior border of the iliac crest and the inferior margin of the last rib mid-axillary plane and the recording was at the end of normal expiration. Hip circumference was measured at the widest level over the greater trochanter. Body mass index (BMI), (kg)/height in m^2^), was used as a measure of total body obesity while waist circumference and waist hip ratio was used as measures of abdominal obesity. Body mass index (BMI) < 18.5 was recorded as underweight, 18.5-24.9 as normal, 25–29.9 as overweight and BMI more than 30 was recorded as obesity. Significant waist circumference was recorded as more than 102 cm (40 inches) for males and more than 88 cm (35 inches) in females. Significant waist to hip ratio was considered abnormal in females with a ratio of >0.8 and males >0.9 [14].

Blood pressure was recorded using a mercury sphygmomanometer (Riester; Jungingen Germany) after the risk factor questionnaire had been filled to ensure that subjects had been seated for at least fifteen minutes. American Heart Association guidelines for measuring blood pressure were used [15]. Three intermittent brachial cuff readings were taken and an average obtained. A random capillary blood sugar (RCBS) was recorded using a glucometer. Participants who had a RCBS >11.1 mmol/l and those with hypertension as per study definition were invited to present themselves to the nearest health facility at a later designated date after an minimal 8 hours overnight fast. A fasting specimen of 5mls blood was drawn from a peripheral vein, preferably the ante-cubital fossa for venous fasting blood sugar (FBS) analysis.

Research assistants underwent training on how to complete the STEPS questionnaire and on anthropometric and blood pressure measurements to ensure standardization. The sphygmomanometers, weighing scales and tape measures were assessed weekly by taking measurements of one person on each of the instruments to ensure they were standardized. Recommended procedures for specimen collection, preparation and storage were followed to minimize pre-analytical errors. Before analysis, all the assays were calibrated according to the manufacturer’s specifications. Commercial controls used to validate the calibrations. Results were transcribed onto data sheets, which were checked by two people to minimize post analytical transcriptional errors.

Hypertension was defined and classified as per the Seventh Report of the Joint National Committee [16] on prevention, detection and treatment of high blood pressure as being systolic BP > = 140 mmHg and/or diastolic BP > = 90 mmHg or use of prescribed antihypertensive medication. Hypertension was classified into pre-hypertension, stage 1 or 2 hypertension as per JNC VII. If the systolic and diastolic pressure readings belonged to different categories, the higher of the two readings was used to assign the blood-pressure stage. Isolated Systolic and Isolated Diastolic Hypertension was defined as per the specified BP cut-offs but with only one category elevated. Participant were diagnosed with diabetes if they had a RCBS level of > = 11.1 mmol/l and a venous FBS of > = 7.0 mmol/l, or had been diagnosed with diabetes or were receiving treatment for diabetes with insulin or oral hypoglycaemic agents. As per the STEPs instrument physical activity (PA) domains and the Global Physical Activity (GPAQ) analysis guide [17], categorical PA levels was coded based on total days and duration of PA and as Total PA in metabolic equivalents of task (METs) minutes per week. Total PA was classified as follows: high as >3000 MET-minutes per week; moderate as 600–3000 MET-minutes per week and low as <600 MET-minutes per week.

Statistical analysis was undertaken using Statistical Products and Service Solutions (SPSS for Windows Ver. 16.0 Chicago, SPSS Inc). Prevalence was age standardised utilising the new WHO World Standard population, direct standardisation method [18]. Associations between the subjects socio-demographic, clinical and laboratory characteristics were examined using chi-square test for the categorical data while for the continuous variables the Student t-test was used to determine statistical significance and Mann Whitney U test used in the analysis where such continuous data is skewed.

Adjusted odds ratios (OR) for the association between hypertension and body mass indices and diabetes were computed with 95% CIs. Binary logistic regression was used to adjust for odds of hypertension associated with s body mass indices and diabetes and the results expressed as OR with 95% confidence intervals (CIs). Multiple linear regression was used to analyse of predictors of elevated systolic and diastolic BP and results expressed as a beta coefficients with 95% CI. Associations were considered significant at the conventional value of p value less than or equal to 0.05.

### Ethical considerations

Study approval was obtained from the Kenyatta National Hospital (KNH) Ethics and Research Committee and the Ministry of Science and Technology. Administrative permission was obtained from the Nairobi City Council and the provincial administration and Kibera community leaders and elders were informed of the study. Verbal consent from head of household and written consent from all eligible adults over the age of 18 yrs was obtained. Follow up care for clinical conditions detected was facilitated by referral to KNH and Mbagathi district Hospital as appropriate.

## Results

We screened 2200 eligible adults from 936 households in eight villages, excluded 139 (38 pregnant women, 50 declined consent and 51 below 18 yrs. of age) and thus enrolled 2061 subjects, representing a 98% response rate. An average of 2.4 adults per household was surveyed. At analysis complete data was available in 2045 subjects with males comprising 50.9%.

Respondent ages ranged from 18 to 90 yrs. with a mean age of 33.4 yrs. (SD 11.6 yrs.); 53.9% were aged 25–44 years, 28.3% were under 25 years and 5.2% were 55 years or older. Literacy level was high with 87% having either a primary or secondary level of educational attainment.

Demographics and behavioural risk factors of the study sample are depicted in Table 1. Current cigarette smokers comprised 13.1%, 84.8% of whom were daily smokers. Alcohol consumption was high with 30% reported to have ever consumed alcohol; 81% of whom consumed alcohol in the previous twelve months with a consumption frequency of 19.1% on a daily basis and 52% between 1–6 days per week; the average and largest number of drinks per sitting being four and six respectfully.

**Table 1:** **Demographic and behavioural risk factors across sex and hypertensive status**

	Overall % (n)	Male % (n)	Female % (n)	P value †	Hypertensive	Normotensive	P value‡
**Age yrs**							
18-24	28.3 (578)	27.7 (291)	28.8 (287)	0.051			
25-34	31.8 (651)	30.1 (316)	33.7 (335)				
35-44	22.1 (452)	22.2 (233)	22.0 (219)				
45-54	12.5 (256)	13.7 (144)	11.3 (112)				
55-64	3.8 (78)	4.8 (50)	2.8 (28)				
65-74	1.2 (25)	1.4 (15)	1.0 (10)				
≥ 75	0.2 (5)	0.1 (1)	0.4 (4)				
18-90	100 (2045)	51.0 (1050)	48.4 (995)				
**Years in school, mean (SD)**	9.3 (3.3)	9.8 (3.2)	8.7 (3.2)	0.000			
**Tobacco smoking**							
Current % (n)	13.1 (269)	22 (231)	3.8 (38)	0.000	17.8 (46)	12.5 (223)	0.018
Smoking daily % (n)	84.8 (228)	89.2 (206)	57.9 (22)	0.000	82.6 (38)	85.2 (190)	0.656
Age started yrs mean (SD)	19.7 (5.5)	19.9 (5.7)	18.6 (3.4)	0.304	21.5 (6.8)	19.4 (5.2)	0.037
Age started range yrs	10-45	10-45	10-24	-	-	-	-
Duration years mean (SD)	16.5 (10)	16.4 (10.2)	17.3 (9.1)	0.696	21.4 (11.3)	15.4 (9.5)	0.001
Pack years median (Q1-Q3)	6 (2.5-10.9)	6 (2.4-10.5)	7.5 (4.4-12.0)	0.303	8.3 (3.9-13.8)	6.0 (2.4-10.5)	0.058
**Alcohol consumption**							
Ever consumed	30.1 (616)	43.2 (454)	16.3(162)	0.000	33.3 (86)	29.7 (529)	0.233
In past 12 months	81.0 (499)	89.6 (407)	56.8 (92)	0.000	76.7 (66)	81.7 (432)	0.281
In past 30 days	76.8 (383)	79.1 (322)	66.3 (61)	0.009	75.8 (50)	77.1 (333)	0.812
Frequency in past 12 months							
Daily	19.1 (95)	22.1 (90)	5.5 (5)	0.008	25.8 (17)	18.1 (78)	0.180
5-6 days/week	14.3 (71)	13.8 (56)	16.5 (15)		7.6 (5)	15.3 (66)	
1-4 days/week	28.7 (143)	27.0 (110)	36.3 (33)		28.8 (19)	28.7 (124)	
1-3 days/week	23.3 (116)	22.9 (93)	25.3 (23)		18.2 (12)	24.1 (104)	
Average number of drinks median (Q1-Q3)	4 (3–6)	4 (3–6)	3 (2–5)	0.087	4 (4–5)	4 (3–6)	0.272
Largest number of drinks/sitting	6 (4.5-9)	6 (5–9)	6 (4–7)	0.026	6 (3–8.5)	6 (5–9)	0.178
**Physical Activity**							
**Work Vigorous**	29.6 (606)	39.1 (411)	19.6 (195)	0.000	24.4 (63)	30.5 (543)	0.050
Days/week median (Q1-Q3)	6 (5–7)	6 (5–7)	6 (3–7)	0.537	6 (5–7)	6 (5–7)	0.116
Hours/day median (Q1-Q3)	8 (3–9)	8 (4–9)	6 (2–8)	0.000	8 (3–9)	8 (4–9)	0.877
**Work moderate**	46.1 (943)	39.1 (411)	53.4 (532)	0.000	43.0 (111)	46.6 (830)	0.295
Days/week median (Q1-Q3)	6 (5–7)	6 (4–7)	7 (5–7)	0.000	6 (5–7)	6 (5–7)	0.420
Hours/day median (Q1-Q3)	6 (3–8)	6 (3–8)	5 (2.1-8.0)	0.000	8 (3–10)	6 (3–8)	0.016
**Travel (walk/cycle)**	77.4 (1583)	80.3 (843)	74.3 (740)	0.001	70.2 (181)	78.5 (1399)	0.003
Days/week median (Q1-Q3)	6 (5–7)	6 (5–7)	6 (4–7)	0.001	6 (5–7)	6 (5–7)	0.148
Hours/day median (Q1-Q3)	1 (0.75-2)	1.3 (0.8-2.0)	1 (0.7-2.0)	0.023	1.3 (0.5-3)	1.0 (0.8-2)	0.480
**Recreational vigorous**	15.2 (311)	21.7 (228)	8.3 (83)	0.000	12.0 (31)	15.7 (280)	0.127
Days/week median (Q1-Q3)	2.0 (1–3)	2.0 (1.0-3.0)	2 (1–3)	0.737	2 (1–5)	2 (1–3)	0.603
Hours/day median (Q1-Q3)	2.0 (0.8-2)	2.0 (1.0-2.0)	1.4 (1–2)	0.142	1.2 (0.5-2)	1.5 (1–2)	0.165
**Recreational moderate**	16.4 (336)	16.9 (177)	16 (159)	0.586	10.5 (27)	17.3 (309)	0.006
Days/week median (Q1-Q3)	3 (1–6)	3.0 (1.0-6.0)	3 (1–6)	0.986	4 (2–7)	3 (1–6)	0.214
Hours/day median (Q1-Q3)	1 (0.7-2)	1.5 (0.8-2.0)	1 (0.7-2)	0.058	2 (0.5-2)	1 (0.8-2)	0.776
Time sitting/reclining hrs mean(range)^+^	4 (2–6)	4.0 (2–6)	4 (2–7)	0.004	5 (2.5-8.0)	4 (2–6)	0.001
METS*, median (Q1-Q3)	10800 (3840–21120)	13474 (5040–24960)	8400 (3168–15630)	0.000	11520 (3840–21110)	10630 (3840–21120)	0.998

^+^Hours per day; sleeping time not included; *METs = metabolic equivalent minutes per week; Q1-Q3 = inter-quartile range; SD = standard deviation.

†P value for sex comparison. ‡P value for Hypertensive status comparison.

### Physical activity

Study subjects demonstrated a high level of physical activity (PA) that was predominantly work and travel related. Vigorous or moderate work related activity was undertaken by 75.7% (male 78.2%, female 73%), at a median duration of 7 hrs for 6 days per week. Walking or cycling as mode of transport was undertaken by 77.4% (male 80.3%, female 74.3%) for a median duration of one hour for 6 days per week. Vigorous work related PA was more common in males whereas moderate work related PA was more common in females. (Table 1) Physical activity data in metabolic equivalents (METS) was available for 87.9% (n 1797) of sample. The median (IQR) total physical activity (PA) in MET-minutes per week was 10800 (3840, 21120) for the entire sample and 13474 (5040, 24960) among males and 8400 (3168, 15630) among females (Table 1). This was indicative of a very high level of total PA in both genders; with 80.2% recording high (>3000) and 17.5% recording moderate (600 to 3000) level of total PA in METs minutes per week.

### Body mass index

Mean (95% CI) Body Mass Index (BMI) was 24.2 (23.9, 24.6) in males and 27.1 (26.6, 27.6) in females (P = 0.000). BMI increased with age in both genders. Majority of males had a normal BMI. However among females 32.2% were overweight (BMI >25%), 26.1% were obese and 38.5% had a normal BMI; thus 58.3% of women were either obese or over-weight. Prevalence of over-weight (BMI >25-29.9) and obesity (BMI ≥30) were significantly higher among women (P = 0.0001 for both BMI categories). Only 5.8% of men and 3.1% of females were underweight.

### Waist circumference

Mean (95% CI) waist circumference (WC) was 81.9 cm (81.3, 82.6) in males and 86.6 cm (85.6, 87.5) in females. WC increased with age in both sexes; however 97.4% of males had a normal WC. The prevalence (95% CI) of WC defined central obesity (WC >88 cm in females & > 108 cm in males) was 41.5% in females and 2.6% in males (P = 0.001).

### Waist-hip ratio

Mean (95% CI) waist-hip ratio (WHR) was 0.89 (0.87, 0.92) in males and 0.85 (0.84, 0.87) in females. Majority of both genders had normal waist-hip ratio (WHR), however 24% of women had an elevated WHR (WHR >0.80) and the prevalence of central obesity as defined by WHR was significantly higher among females (P = 0.001 ). All measures of body mass showed a statistically significant increase with age among females (P = 0.000); and the same were evident among male subjects (P = 0.000) except for WHR that did not show a statistically significant increase with age (P = 0.269).

### Blood pressure

**Figure 1:**
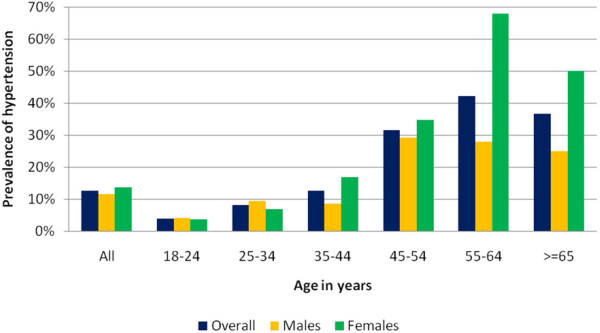
**Prevalence of hypertension across age and sex.**

The crude prevalence (95% CI) of hypertension (BP ≥ 140/90 mmHg or taking prescribed antihypertensive medication) was 12.6% (11.2, 14.1); 11.7% (9.7, 13.6) in males and 13.7% (11.5, 15.8) in females (P = 0.166). Prevalence increased with age and was significantly higher among females in the age strata of 35–44 yrs. (p = 0.008) and 55-64 yrs (P = 0.001) (Figure 1). The new WHO Standard population age standardised prevalence of hypertension (95% CI) was 22.8% (20.7, 24.9) in the entire sample and 23.2% (20.4, 26.1) in analysis restricted to those over 35 years of age. Only 20% (53/258) were aware of their hypertensive status and among the known and drug treated hypertensive subjects 49% (26/53) had controlled hypertension at time of survey (BP < = 140/90 mmHg.).

Only 30.9% (631/2045) of study subjects reported ever having had a BP measured by health workers and 22.3% (141/631) having been told that their BP was elevated; 95.7% (135/141) having been informed so in the twelve months prior to interview. Of those informed of an elevated BP in the past year 39.5% (53/135) had been prescribed antihypertensive therapy and were on the therapy in the two weeks prior to interview. The proportions advised on non-pharmacological measures for BP control were: 40% salt restriction, 28.9% increased physical activity, 25.2% weight reduction and 17% smoking cessation. Regarding alternative therapies 17% had ever seen a traditional healer and 11.9% were on current herbal or traditional remedies for the treatment of raised BP.

The mean (95% CI) systolic blood pressure (SBP) among males and females was 124 mmHg (123.1, 124.8) and 123.5 mmHg (122.7, 124.4) respectively. The mean (95% CI) diastolic blood pressure (DBP) among males and females was 77.5 mmHg (76.9, 78.1) and 76.9 mmHg (76.2, 77.5) respectively. SBP & DBP increased with age in both sexes, with no gender differential. For both SBP & DBP females had lower BP reading in the younger age strata and higher reading in the older age strata, with the transition occurring in the 35-44 yr age strata.

Among those subject with screening detected blood pressure, that was higher than JNC defined optimal blood pressure (BP >120/80 mmHg) (i.e. excluding those with known hypertension and or on treatment), 59.3% had pre-hypertension (61.8% male; 56.6% females) and 6.2% had Stage 1 and 4.1% Stage 2 Hypertension (Table 2).

**Table 2:** **Prevalence of hypertension, high blood pressure, pre-hypertension and hypertension stages**

	Overall n (%)	Male n (%)	Female n (%)	P value
**Hypertension**				
Yes	258 (12.6)	122 (11.6)	136 (13.7)	0.165
No	1787 (87.4)	928 (88.4)	859 (86.3)	
**Blood Pressure**				
Normal	604 (30.4)	288 (27.9)	316 (33.1)	0.073
Pre-Hypertension	1178 (59.3)	637 (61.8)	541 (56.6)	
High Blood Pressure*	205 (10.3)	106 (10.3)	99 (10.4)	
Stage 1	124 (6.2)	65 (6.3)	59 (6.2)	
Stage 2	81 (4.1)	41 (4.0)	40 (4.2)	

*High Blood Pressure category excludes subjects known to be hypertensive and on treatment at survey.

In the entire sample, the crude prevalence (95% CI) of Isolated Systolic Hypertension (ISH) and Isolated Diastolic Hypertension (IDH) was 1.6% (1.1, 2.2); [males 1.5% (0.8, 2.2) females 1.8% (1.2,2.7)], and 2.7% (2.0, 3.4) [males 2.9% (1.9, 4.1) females 2.5% (1.6, 3.5)] respectively. The combined prevalence of ISH and IDH in the entire sample was 6.0% (4.9, 7.0); 5.9% (4.1, 7.4) in males and 6.1% (4.6, 7.7) in females. The age strata of 55–64 yrs. demonstrated a higher female prevalence of high blood pressure and combined systolic and diastolic hypertension (Additional file 1). Additional file 2 depicts the crude prevalence of high blood pressure and isolated forms of hypertension in those with high blood pressure (ie excluding subjects with known hypertension and on treatment at survey).

### Risk factors distribution by hypertension status

Behavioural cardiovascular risk factors distribution by hypertensive status are depicted in Table 1. Compared to normotensive, a larger proportion of hypertensive subjects were current Smokers (17.8%; 12.5%; P = 0.018), had commenced smoking at older age (21.4 yrs.; 15.4 yrs; P = 0.037) and had a longer duration of smoking (8.3 yrs, 6.0 yrs, P = 0.001). Alcohol consumption levels among hypertensives was high and equal to that of normotensives and the entire sample. Hypertensives demonstrated a similar pattern of high level of physical activity (PA) as normotensives. The median total PA (Q1-Q3)) in MET-minute per week in hypertensive subjects was 11520 (3840, 21110) and 10630 (3840, 21120) in normotensive subjects (P = 0.998).

Categories of body mass indices stratified on hypertensive status are depicted in Table 3. Compared to normotensive, the age, sex and alcohol adjusted OR (95% CI) of a hypertensive being obese was 2.9 (1.9, 4.4), of being over-weight 1.8 (1.2, 2.5) and of having waistline defined central obesity 2.4 (1.6, .6); with no significant gender differences. An elevated waist–hip ratio was not significantly associated with an increased likelihood of hypertension (OR 1.3; 95% CI 0.8, 2.0).The proportion of hypertensive subjects who also had diabetes was 14% (n 36). Compared to normotensive, hypertensive subjects were 4.4-fold (95% CI 2.4-8.3) more likely to have diabetes. The odds of a female hypertensive having diabetes was 2.9 fold (95% CI 1.0, 8.3) that of a male hypertensive though this was of borderline statistical significance (P = 0.049). The odds of diabetes in the normotensive did not demonstrate a gender propensity.

**Table 3:** **Body Mass Indices and Diabetes Distribution by Hypertension Status**

Variable	Hyper-tensive % (n)	Normo-tensive % (n)	Odds ratio (95% CI)	p value	Age adjusted odds ratio (95% CI)	p value	Age-sex adjusted odds ratio (95% CI)	p value	Age-sex-smoking-alcohol use adjusted odds ratio (95% CI)	p value
**BMI (n = 2037)**										
≥ 30	34.0 (87)	13.8 (245)	4.4 (3.1-6.2)	0.000	3.2 (2.3-4.6)	0.000	3.2 (2.2-4.7)	0.000	2.9 (1.9-4.4)	0.000
>25-29.9	33.6(86)	28.4 (506)	2.1 (1.5-2.9)	0.000	1.7 (1.2-2.4)	0.002	1.7(1.2-2.4)	0.002	1.8(1.2-2.5)	0.003
18.5-25	29.7 (76)	53.0 (943)	Ref		Ref		Ref		Ref	
<18.5	2.7 (7)	4.8 (85)	1.0 (0.5-2.3)	0.956	1.1 (0.5-2.4)	0.883	1.1 (0.5-2.4)	0.884	1.2 (0.5-2.7)	0.745
**Waist Circ. (n = 2032)**										
Elevated	39.6 (101)	18.9 (335)	2.8 (2.1-3.7)	0.000	2.1 (1.6-2.8)	0.000	2.4 (1.6-3.5)	0.000	2.4 (1.6-3.6)	0.000
Normal	60.4 (154)	81.1 (1440)	Ref		Ref		Ref		Ref	
**Waist Hip Ratio (n 2027)**										
High	22.0 (56)	12.0 (212)	2.1 (1.5-2.9)	0.000	1.6 (1.1-2.2)	0.012	1.4 (1.0-2.1)	0.074	1.3 (0.8-2.0)	0.273
Normal	78.0 (198)	88.0 (1559)	Ref		Ref		Ref		Ref	
Diabetes	14.0 (36)	1.7 (30)	9.5 (5.7- 15.7)	0.000	5.1 (3.0-8.7)	0.000	4.9 (2.9-8.5)	0.000	4.4 (2.4-8.3)	0.000
No Diabetes	86.0 (222)	98.3 (1758)	Ref		Ref		Ref		Ref	

BMI = body mass index. Categories: ≥30 Obese; >25-29.9 Over-weight <18.5 Under-weight.

Waist Circ = Waist Circumference. Categories: Elevated Male >102 cm; Female >88 cm.

Waist Hip Ratio High: >0.90 males and 0.80 in females.

In a multivariable linear regression model, the association between age and WC with blood pressure (BP) showed that per decade increase in age SBP increased by 2.6 mmHg (1.86 mmHg males; 3.6 mmHg females) and DBP increased by 2.2 mmHg (1.6 mmHg males; 3.0 mmHg females).Similarly per five centimetre increase in WC, SBP increased by0.50 mmHg (0.78 mm Hg males & 0.35 mmHg females) and DBP increased by 0.40 mmHg (0.56 mmHg male; 0.32 mmHg females). Per unit increase in BMI, SBP increased by 0.2 mmHg in both gender, and DBP increased by 0.08 mmHg (0.12 mmHg males, 0.06 mmHg females).

## Discussion

This study of adults living in the largest urban slum in Kenya shows high age adjusted prevalence of hypertension with close to quarter (23%) classified as hypertensive and 60% as pre-hypertensive. Majority (80%) were undetected and only one in three study subjects had ever undergone blood pressure screening suggesting a low overall awareness of hypertension. Hypertension prevalence increased with age and tended to be higher in women in the older age categories. Our reported hypertension prevalence is consistent with that of recently published reports in rural and urban non-slum African studies [19–21]. Studies on the prevalence of hypertension in Kenya are sparse and until recently none had been undertaken among slum residents [8, 9, 22]. A 2011 report from Old Town Mombasa reports an adjusted prevalence of 32% [22]. No regional studies have been undertaken among slum dwellers. Two studies in Kenya have reported on hypertension among poor urban slum residents [23, 24]. Of these one was among a non-probability sample of Kibera slum resident and reported an unadjusted hypertension prevalence of 13% [24]. The other utilised cluster sampling and reported a WHO standardised population prevalence of 18.4%, inclusive of an 80% screen detection rate [23].

Our study subjects were homogeneous with regards to high levels of travel or work related physical activity and important high levels of harmful alcohol intake. Ten percent were current smokers. Majority of males had a normal body mass measurement, however females showed a high prevalence of excess body mass with one-third being obese or overweight and 40% having central obesity.

Correlates of hypertension included advancing age, overweight, general and central obesity which were associated with 2–3 fold increased likelihood of hypertension, affirming that these are risk factor for development of primary hypertension in this population. These associations are well established and have been described in SSA [21], and in particular were similarly demonstrated in a recent Nairobi slum survey report [23].

One out of every seven hypertensive was found to be diabetic with an almost five fold likelihood of a hypertensive being diabetic. In a related publication we reported age adjusted diabetes prevalence of 5%, with one in two persons with diabetes being hypertensive and a threefold likelihood of person with diabetes being hypertensive [25]. The association between these cardiovascular NCD risk factors of diabetes and hypertension is well established, and compounds total cardiovascular risk [26]. This finding points to the possibility of using blood pressure measurement as a first step in cardiovascular non-communicable disease screening in community and resource poor settings.

Our findings of high prevalence of predominantly undetected hypertension and pre-hypertension in association with behavioural/physiological risk factors (of alcohol and high body weight and diabetes) in this poor urban slum community, with high levels of physical activity, point to an early social transition of cardiovascular disease in the urban poor and the need for primary and secondary prevention strategies through lifestyle interventions. Furthermore an increased and sustained awareness of the burden of hypertension among the public and health personnel is required. This will assist in the detection, treatment and control of hypertension and associated risk factors.

A detailed study of the dietary practises contributing to excess body weight and salt intake, in a community with limited opportunity for increased physical activity, is paramount and should be undertaken alongside that of the culturally acceptable and desired body shapes among Africans. This in particular among females who have a greater burden of obesity and have been shown in other studies to have a lack of awareness of the presence and hazards of excess body weight and thus little desire for change [27].

### Strengths and limitations

To the best of our knowledge this is among the first published urban slum NCD survey report from Kenya, whose strengths and limitations have been discussed in a related publication. [25]. In brief our strengths include reported age standardised prevalence, utilising standard survey methods that allow for comparability across studies and regions. By sampling an average of 2.4 adults per household we do not expect that over representation of family size impacted our results. We acknowledge the random walk method in household survey as not optimal for obtaining a probabilistic sample. The availability of dietary data, including salt intake, and socio-economic and lipid profile data would have strengthened our findings. The limitations of self reported physical activity and the inherent limitation of a cross-sectional study design in prohibiting causal interpretation are acknowledged. Our instruments standardisation method for weight and BP, were suited to a field slum study circumstances, however could have potentially introduced a non-differential bias. In clinical practice, a diagnosis of hypertension requires multiple measurements on several occasions. We took a single occasion measurement and therefore, the prevalence of hypertension found in our survey may represent an overestimation.

## Conclusion

In conclusion the age adjusted prevalence of hypertension is high, even among the urban poor living in a low income country, and is significantly correlated with overweight and obesity that is not uncommon among this community. Majority of the hypertensive subjects are undetected and therefore unaware of the risks they face; a recognised major impediment to control of hypertension. This points to the need for greater awareness of hypertension in the general population and health care givers. In tandem, within the health system greater emphasis needs to be placed on detection, treatment and control of high blood pressure. Preventive efforts targeting behavioural lifestyle changes, at community level, to curtail these risk factors at population and individual level is urgently required. Reducing these risk factors will not only have an effect on hypertension, but will also have wide-reaching beneficial effects for other chronic non-communicable diseases.

## Electronic supplementary material

Additional file 1: **Prevalence of high blood pressure and isolated forms of hypertension by age and sex.** (PDF 105 KB)

Additional file 2: **Prevalence of high blood pressure and isolated forms of hypertension in those with high blood pressure by age and sex.** (PDF 24 KB)
