# Supplementary material for: Prevalence of hypertension and associated cardiovascular risk factors in an urban slum in Nairobi, Kenya: A population-based survey
Source: BMC Public Health. 2014 Nov 18;14:1177. doi: 10.1186/1471-2458-14-1177 (PMC4246542; doi:10.1186/1471-2458-14-1177)
Supplement: Supplementary file 1 — Additional file 1: Prevalence of high blood pressure and isolated forms of hypertension by age and sex. (PDF 105 KB) [file 12889_2014_7257_MOESM1_ESM.pdf]

Additional File 1

Table : Prevalence of High Blood Pressure and Isolated forms of Hypertension by Age and Sex.

AGE CATEGORY	OVERALL			MALE			FEMALE			P value
	**n**	**%**	**95 % CI**	**n**	**%**	**95 % CI**	**N**	**%**	**95% CI**	
High Blood Pressure (HBP)										
18-24	21	3.6	(2.2, 5.4)	12	4.1	(2.0, 6.7)	9	3.2	(1.4, 5.3)	0.536
25-34	44	6.9	(4.9, 8.9)	28	8.9	(5.8, 12.0)	16	4.9	(2.5, 7.4)	0.043
35-44	41	9.4	(6.8, 12.3)	18	7.9	(4.6, 11.5)	23	11.2	(6.8, 15.7)	0.232
45-54	63	26.6	(21.2, 2.3)	33	24.4	(17.4, 32.6)	30	29.4	(20.4, 38.3)	0.391
55-64	26	36.6	(25.8, 9.3)	11	23.4	(11.8, 36.2)	15	62.5	(42.9, 82.1)	0.001
>=65	10	34.5	(16.1, 3.1)	4	25.0	(4.8, 50.0)	6	46.2	(14.9, 75.0)	0.270
18-90	205	10.3	(9.0, 11.7)	106	10.4	(8.7, 12.4)	99	10.3	(8.5, 12.3)	0.957
Isolated Systolic Hypertension										
18-24	6	1.0	(0.3, 2.0)	5	1.7	(0.4, 3.4)	1	0.4	(0.0, 1.1)	0.216
25-34	3	0.5	(0.0, 1.1)	2	0.6	(0.0, 1.6)	1	0.3	(0.0, 1.0)	0.616
35-44	4	0.9	(0.2, 2.0)	2	0.9	(0.0, 2.3)	2	1.0	(0.0, 2.5)	1.000
45-54	11	4.6	(2.2, 7.5)	4	3.0	(0.4, 6.4)	7	6.9	(2.0, 12.6)	0.214
55-64	5	7.0	(1.6, 13.5)	1	2.1	(0.0, 7.1)	4	16.7	(3.6, 34.5)	0.042
>=65	3	10.3	(0.0, 23.8)	1	6.3	(0.0, 20.0)	2	15.4	(0.0, 35.7)	0.573
18-90	32	1.6	(1.1, 2.2)	15	1.5	(0.8, 2.2)	17	1.8	(1.0, 2.7)	0.567
Isolated Diastolic Hypertension										
15-24	8	1.4	(0.5, 2.4)	2	0.7	(0.0, 1.8)	6	2.1	(0.7, 4.1)	0.173
25-34	17	2.7	(1.4, 4.0)	11	3.5	(1.6, 5.8)	6	1.8	(0.6, 3.5)	0.187
35-44	15	3.5	(1.8, 5.3)	7	3.1	(0.9, 5.4)	8	3.9	(1.4, 6.7)	0.630
45-54	11	4.6	(2.0, 7.3)	8	5.9	(2.2, 10.3)	3	2.9	(0.0, 6.5)	0.359
55-64	2	2.8	(0.0, 7.6)	1	2.1	(0.0, 7.4)	1	4.2	(0.0, 14.3)	1.000
>=65	1	3.4	(0.0, 11.4)	1	6.3	(0.0, 19.0)	0	0.0	-	1.000
18-90	54	2.7	(2.0, 3.4)	30	2.9	(1.9, 4.1)	24	2.5	(1.6, 3.5)	0.584
Systolic and Diastolic Hypertension										
15-24	7	1.2	(0.3, 2.2)	5	1.7	(0.4, 3.4)	2	0.7	(0.0, 1.8)	0.450
25-34	24	3.8	(2.3, 5.4)	15	4.8	(2.6, 7.1)	9	2.8	(1.1, 4.7)	0.174
35-44	22	5.1	(3.1, 7.3)	9	3.9	(1.6, 6.7)	13	6.3	(3.2, 9.7)	0.253
45-54	41	17.3	(13.0, 2.1)	21	15.6	(9.6, 22.0)	20	19.6	(12.1, 27.9)	0.414
55-64	19	26.8	(15.9, 7.7)	9	19.1	(8.6, 30.4)	10	41.7	(20.8, 61.9)	0.043
>=65	6	20.7	(6.9, 37.9)	2	12.5	(0.0, 33.3)	4	30.8	(7.1, 60.0)	0.364
18-90	119	6.0	(4.9, 7.0)	61	5.9	(4.1, 7.4)	58	6.1	(4.6, 7.7)	0.888

High Blood Pressure (HBP)
