# Supplementary material for: Prevalence of hypertension and associated cardiovascular risk factors in an urban slum in Nairobi, Kenya: A population-based survey
Source: BMC Public Health. 2014 Nov 18;14:1177. doi: 10.1186/1471-2458-14-1177 (PMC4246542; doi:10.1186/1471-2458-14-1177)
Supplement: Supplementary file 2 — Additional file 2: Prevalence of high blood pressure and isolated forms of hypertension in those with high blood pressure by age and sex. (PDF 24 KB) [file 12889_2014_7257_MOESM2_ESM.pdf]

Additional File 2

TABLE : Prevalence of High Blood Pressure and Isolated forms of Hypertension in those with High Blood Pressure by Age and Sex.

AGE CATEGORY				MALE			FEMALE			P value
	N	%	95 % CI	n	%	95 % CI	n	%	95% CI	
Isolated systolic hypertension by age group in those with HBP										
15-24	6	28.6	(10.5, 47.8)	5	41.7	(12.5, 72.7)	1	11.1	(0.0, 40.0)	0.216
25-34	3	6.8	(0.0, 14.3)	2	7.1	(0.0, 17.9)	1	6.3	(0.0, 22.2)	0.616
35-44	4	9.8	(2.2, 20.0)	2	11.1	(0.0, 30.7)	2	8.7	(0.0, 23.1)	1.000
45-54	11	17.5	(8.8, 27.7)	4	12.1	(2.9, 25.0)	7	23.3	(8.7, 39.3)	0.214
55-64	5	19.2	(4.3, 35.5)	1	9.1	(0.0, 30.0)	4	26.7	(6.3, 50.0)	0.042
>=65	3	30.0	(0.0, 62.5)	1	25.0	-	2	33.3	-	0.573
18-90	32	15.6	(10.3, 21.0)	15	14.2	(7.5, 20.9)	17	17.2	(10.3, 24.5)	0.567
Isolated diastolic hypertension by age group in those with HBP										
15-24	8	38.1	(16.7, 61.5)	2	16.7	(0.0, 42.8)	6	66.7	(33.3, 100.0)	0.173
25-34	17	38.6	(24.3, 53.1)	11	39.3	(21.4, 58.3)	6	37.5	(12.7, 62.5)	0.187
35-44	15	36.6	(22.6, 52.0)	7	38.9	(15.8, 62.5)	8	34.8	(15.0, 55.6)	0.630
45-54	11	17.5	(7.7, 27.4)	8	24.2	(10.5, 40.0)	3	10.0	(0.0, 23.5)	0.359
55-64	2	7.7	(0.0, 20.0)	1	9.1	(0.0, 28.6)	1	6.7	(0.0, 25.0)	1.000
>=65	1	10.0	(0.0, 33.3)	1	25.0	-	0	0.0	-	1.000
18-90	54	26.3	(20.6, 32.4)	30	28.3	(20.4, 37.1)	24	24.2	(16.0, 33.3)	0.584
Systolic and diastolic hypertension by age group in those with HBP										
15-24	7	33.3	(13.7, 53.3)	5	41.7	(14.3, 71.4)	2	22.2	(0.0, 50.0)	0.642
25-34	24	54.5	(39.5, 69.2)	15	53.6	(34.5, 72.4)	9	56.3	(30.0, 81.8)	0.864
35-44	22	53.7	(38.2, 68.8)	9	50.0	(26.7, 73.3)	13	56.5	(35.0, 77.3)	0.678
45-54	41	65.1	(52.8, 76.4)	21	63.6	(45.5, 80.6)	20	66.7	(48.5, 83.3)	0.801
55-64	19	73.1	(55.2, 90.0)	9	81.8	(55.6, 100.0)	10	66.7	(41.7, 90.0)	0.658
>=65	6	60.0	(28.6, 90.0)	2	50.0	-	4	66.7	-	1.000
18-90	119	58.0	(51.7, 64.8)	61	57.5	(47.7, 67.0)	58	58.6	(49.5, 67.7)	0.880

High Blood Pressure (HBP)
